# Adnexal Torsion During Pregnancy in a Surrogate Patient Post-tubal Ligation: A Case Report

**DOI:** 10.7759/cureus.66529

**Published:** 2024-08-09

**Authors:** Tayler Mackie, Anna Woodham, Abdelrahman Yousif

**Affiliations:** 1 Department of Obstetrics and Gynecology, Paul L. Foster School of Medicine, Texas Tech University Health Sciences Center El Paso, El Paso, USA

**Keywords:** bilateral tubal ligation, pregnancy, laparoscopy, ovarian cyst, surrogacy, pelvic pain, adnexal torsion

## Abstract

Adnexal torsion during pregnancy is rare and is complicated by ambiguous symptoms and often nonspecific imaging findings. Differential diagnoses of torsion include a ruptured ovarian cyst, tubo-ovarian abscess, and appendicitis. A low threshold for the recommended surgical laparoscopy is necessary to avoid delayed diagnosis and fetal or maternal complications. We present a case of a 30-year-old woman at 10 weeks gestation as a surrogate carrier, admitted for progressive, sharp lower right quadrant abdominal pain. On presentation, she was afebrile and vitally stable, with moderate leukocytosis and elevated inflammatory markers. Transvaginal ultrasound showed a 6 x 6 cm adnexal mass/cyst, without ovarian vascular compromise, in addition to a tubular structure indicating possible hydrosalpinx. Initially, her presenting symptoms partially* *resolved following antibiotics and analgesics, which led us to consider a tubo-ovarian abscess as the culprit. However, upon a recurrence of pain, we proceeded with a diagnostic laparoscopy, with a high suspicion of ovarian torsion. A right adnexal torsion and paratubal cyst were identified; detorsion with preservation of adnexa and cystectomy was performed, with resolution of the pain in the postoperative period. This case underscores the importance of identifying multiple risk factors and complex clinical scenarios for ovarian torsion in premenopausal patients in the context of surrogate pregnancies following tubal ligation. Our findings contribute to the existing literature by emphasizing the need for a high index of suspicion for adnexal torsion, as it is imperative to prevent complications and ensure prompt surgical intervention.

## Introduction

Adnexal torsion is the fifth most common gynecologic emergency [1]. Adnexal masses involving the ovaries and fallopian tubes are the most common causes of adnexal torsion, especially benign functional ovarian cysts and benign teratomas [2,3]. Adnexal torsion in pregnancy is most often caused by corpus luteum cysts. Other risk factors for adnexal torsion include previous tubal ligation and pregnancy [4]. Ovarian torsion risk rises to about 15% during pregnancy and is difficult to diagnose partly due to the overlap of presenting symptoms and signs [3]. For example, adnexal torsion patients commonly present with abdominal pain, nausea, and vomiting, which are often present in pregnant patients. Ovarian torsion is a feared complication in pregnancy, as it is associated with maternal and fetal morbidities and mortality [5]. Transabdominal ultrasound (US) is the diagnostic modality of choice for adnexal torsion. Enlarged ovaries and absent blood flow on Doppler studies are suggestive signs of adnexal torsion [6]. Prompt diagnosis and treatment are essential to preserve ovarian function and future fertility. With suspicious presenting symptoms and suggestive sonographic signs, diagnostic laparoscopy should be performed to confirm the diagnosis. Current recommendations suggest a minimally invasive surgical approach with detorsion and preservation of adnexal structures, regardless of the appearance of the ovary [7]. Here, we present a unique case of adnexal torsion during pregnancy post-bilateral tubal ligation, presenting with acute abdominal pain to the Emergency Department.

## Case presentation

A 30-year-old gravida 6, para 3, at 10 weeks gestation, presented to the Emergency Department due to a four-day history of progressive abdominal pain. She is also a gestational surrogate. She reported that the pain began in the right lower abdominal quadrant and started to radiate to her right flank and diffusely throughout her abdomen. She described her pelvic pain as feeling sore, along with intermittent bouts of sharp pain rated 8 to 9 out of 10. She noted chills but denied fevers, nausea, vomiting, or vaginal bleeding. The patient was afebrile, and all other vital signs remained stable.

Her past medical history was negative, aside from a urinary tract infection earlier in the pregnancy, which was treated with cephalexin. She underwent a bilateral tubal ligation in 2016. Her prenatal labs and screening were all within normal limits. Upon initial physical examination, the patient was alert and in moderate distress. Upon abdominal examination, diffuse, severe abdominal tenderness was noted. On admission, a pelvic examination was unremarkable, without masses or lesions. Significant diffuse tenderness was noted on bimanual examination.

**Figure 1:**
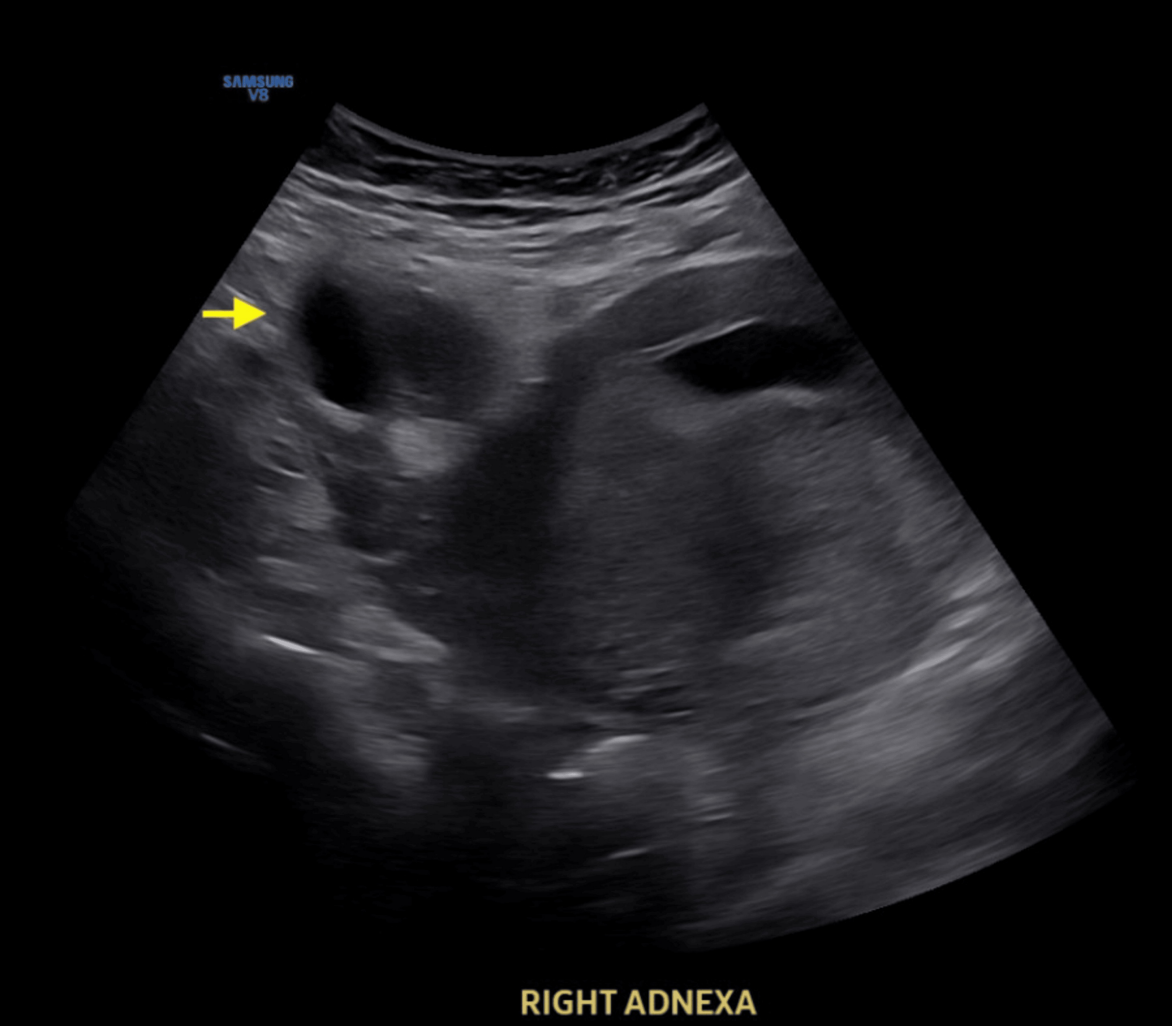
Transabdominal ultrasound of a right anechoic cyst (arrow) measuring 4.9 x 3.8 x 6.1 cm adjacent to the right ovary.

**Figure 2:**
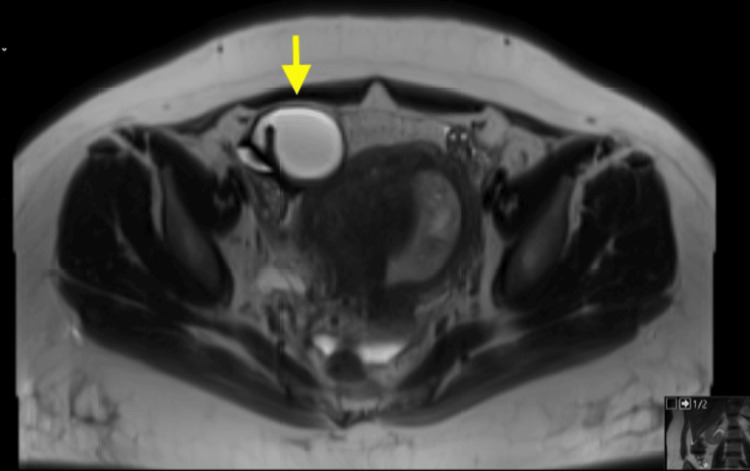
Axial T2 weighted pelvic MRI without contrast identifying a right adnexal cyst (yellow arrow) measuring 4 cm.

On the day of arrival, a transvaginal ultrasound (TVUS) showed an anechoic tubular structure adjacent to the right adnexa with concern for a hydrosalpinx, without evidence of tubo-ovarian abscess (TOA) (Figure 1). Next, magnetic resonance imaging (MRI) of the pelvis without contrast was performed to rule out appendicitis. MRI was negative for appendicitis but confirmed a hydrosalpinx measuring 4 cm transversely, with an adjacent adnexal cyst (Figure 2). Initial laboratory findings were positive for leukocytosis, with a WBC count of 15.45 × 10³/µL and a neutrophil shift of 13.33 × 10³/µL. Initial markers of inflammation were elevated, with an erythrocyte sedimentation rate (ESR) of 42 mm/hr and a C-reactive protein (CRP) level of 8.14 mg/dL. 

Due to the presence of a right adnexal cystic structure with hydrosalpinx and layering debris visualized on MRI, leukocytosis, and four days of subacute abdominal pain, there was a high index of suspicion for TOA versus adnexal torsion. Other differential diagnoses considered on presentation included appendicitis, ovarian cyst rupture, and nephrolithiasis. Each was ruled out clinically via history and physical examination, as well as the absence of diagnostic signs on pelvic MRI. This included the visualization of minimal free pelvic fluid, a normal-appearing appendix, and the absence of renal calculi.

The decision was made to admit the patient for antibiotic treatment and serial physical exams to monitor her condition. The patient was started on IV gentamicin 5 mg/kg every 24 hours and IV clindamycin 900 mg every eight hours for 48 hours, following the recommendations of the Centers for Disease Control and Prevention (CDC) for managing pelvic inflammatory disease in pregnancy. Following antibiotic treatment, the patient reported pain improvement with partially resolved symptoms. However, on day 3 of admission, the patient required increased doses of pain medications. Given the worsening of her pain and the increased medication requirements that day, our team recommended performing a diagnostic laparoscopy to investigate the source of her pain further and to rule out adnexal torsion. 

**Figure 3:**
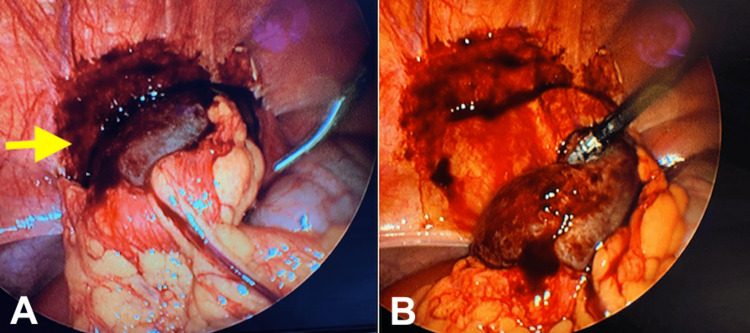
The left pane (A) demonstrates a paratubul cyst (arrow) measuring 4.5 x 4.3 x 1.6 cm adherent to the right anterior abdominal wall. The right pane (B) visualizes the cyst dissected from the anterior abdominal wall.

**Figure 4:**
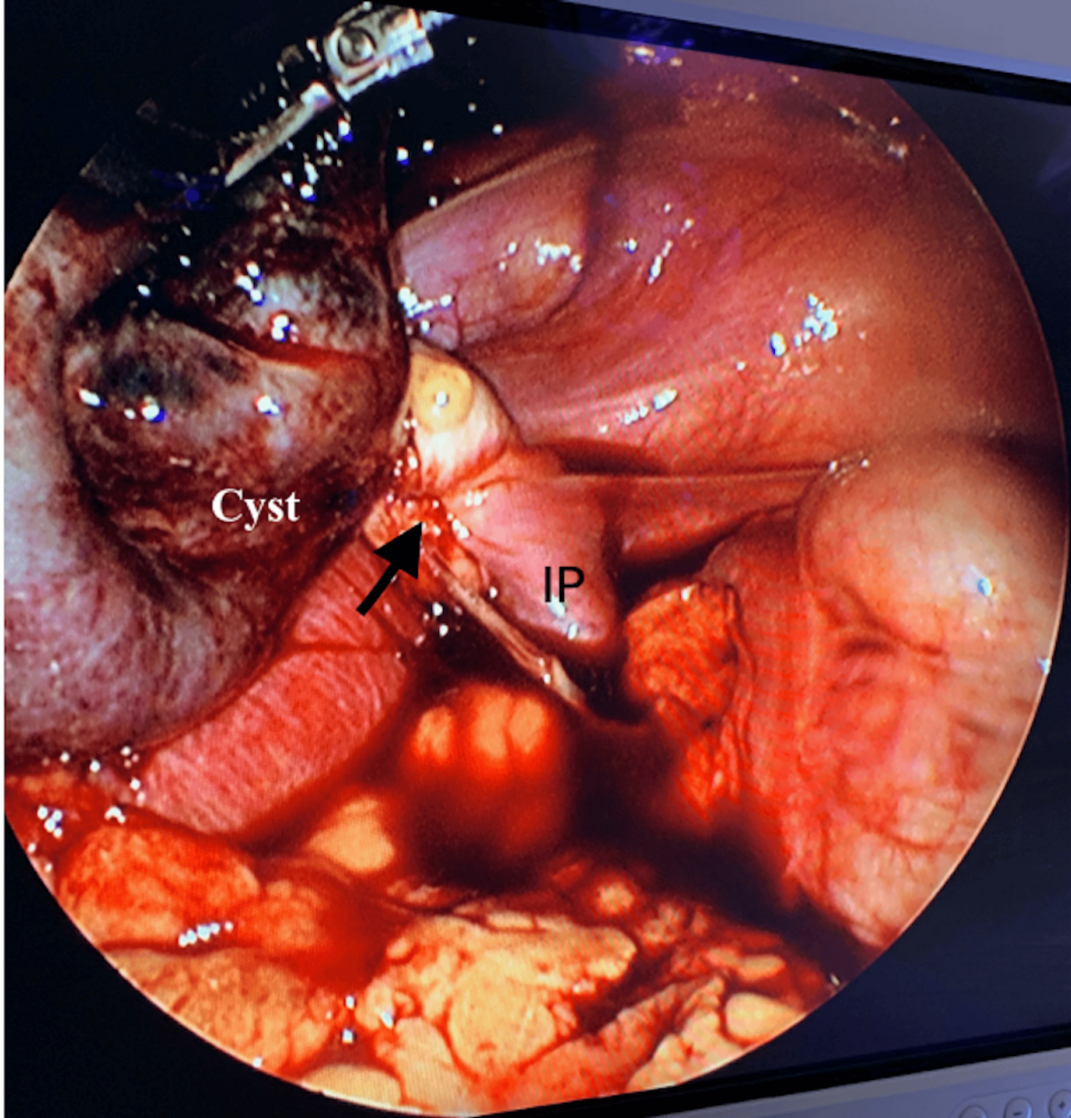
Laparoscopic evidence of a 4.5 x 4.3 x 1.6 cm paratubal cyst adhered to the abdominal wall with a 360-degree right infundibulopelvic (IP) ligament torsion (arrow). No vascular compromise was appreciated.

During the laparoscopy, visualization revealed moderate adhesive disease surrounding the right fallopian tube, with a large cystic structure adhered to the right anterior abdominal wall. A right paratubal cyst, measuring 4.5 x 4.3 x 1.6 cm, with 360-degree right infundibulopelvic ligament torsion, was freed from the abdominal wall and transected (Figure 3). The surgeons recognized a right ovarian torsion but had no concern for ovarian vascular compromise (Figure 4). The patient was vitally stable, with reassuring fetal heart tones following cystectomy and detorsion. Upon histopathological review of the cyst, a hemorrhagic simple paratubal cyst was confirmed. The patient was discharged the following day after meeting post-operative milestones. Her pregnancy was complicated by premature preterm rupture of membranes at 33 weeks, and she had a successful vaginal delivery at 34 weeks after augmentation of labor.

## Discussion

Ovarian torsion is a gynecologic emergency that lacks a pathognomonic presentation, underscoring the importance of a comprehensive evaluation through clinical presentation, a thorough history, and a physical examination. Ovarian torsion is caused by the rotation of the adnexa around its pedicle, resulting in vascular and lymphatic flow interference [5]. In pregnancy, the etiology of ovarian torsion is often due to a corpus luteal cyst and is more prevalent in the first trimester. Of note, our patient presented to us in the first trimester.

The primary symptom of torsion is acute onset unilateral abdominal pain, with or without fever, nausea, and vomiting [4]. Among pregnant patients, we hypothesize that possible factors leading to a longer clinical course of four-day progressive abdominal pain in our patient might have occurred due to anatomical changes, hormonal fluctuations, and misinterpretation of symptoms. Other differential diagnoses important to consider in patients presenting with severe lower abdominal pain, with or without radiation, are appendicitis, TOA, ruptured ovarian cyst, ectopic pregnancy, and nephrolithiasis. In our case, appendicitis and renal colic were eliminated promptly by diagnostic imaging. An initial physical exam also aided in narrowing the differential, based on the patient being afebrile with a negative McBurney's point.

On US imaging, common findings include enlarged adnexa, ovarian stromal edema, the whirlpool sign, and free fluid in the pelvis [6]. In this case, the first US imaging demonstrated a possible hydrosalpinx, which MRI confirmed. Small free fluid, mostly in the cul-de-sac, was also seen. No impedance of blood flow was visualized on the TVUS Doppler study. As discussed by Sasaki and Miller and by Moro et al., the absence of Doppler signals is relatively common and does not exclude torsion [4,6]. While US imaging is the mainstay in diagnosis, CT and MRI are additional modalities that are useful. During pregnancy, MRI is a safer option. Laboratory results are often non-specific and may show mild leukocytosis in 27% to 50% of patients, which was also observed in this case [8]. Inflammatory markers, like CRP, may also be elevated in ovarian torsion and other common pathologies, i.e., appendicitis and TOA [9]. In a study by Ribak et al., CRP levels above 49.3 mg/dL were predictive of TOA [10]. Our patient's CRP level was 13 mg/dL at its highest value, in comparison.

Paratubal cysts are among the most common pathological conditions causing adnexal torsion [4]. Cyst size associated with a higher risk of torsion is variable. One study found that 89% of patients with an ovary greater than 5 cm underwent torsion, with a mean size of 9.5 cm [1]. A retrospective study by Yen et al. found that 14% of pregnant women with an adnexal mass greater than 4 cm experienced an episode of adnexal torsion [3]. In our case, the cyst measured just over 4 cm, making it a probable cause of torsion. Tubal ligation is another known risk factor for ovarian torsion due to the presence of postsurgical adnexal adhesions [1,11]. Moderate adhesive disease was found on the right fallopian tube in our patient, highlighting another possible underlying risk factor for torsion in this patient. Though case reports of isolated fallopian torsion have been reported following sterilization, including one with a tubal cyst similar to ours [12-14], our case of ovarian torsion follows a more classic presentation. We hypothesize that the patient's risk factors were cumulative, including a history of tubal surgery with evidence of adhesive disease, pregnancy status, and cyst size. Additionally, cystic adhesion to the abdominal wall may have occurred following torsion due to an increased inflammatory response from pregnancy, decreased anatomic proximity (uterus enlargement), and peritoneal irritation. 

Laparoscopic surgery was performed in this case, which is in line with current literature supporting this approach over laparotomy [4]. When compared to non-pregnant women, a laparoscopic approach showed no difference in outcomes and was associated with the additional benefits of fewer complications, reduced narcotic requirements, and improved recovery times [15].

## Conclusions

While ovarian torsion during pregnancy is uncommon, it is a condition requiring emergent intervention. To our knowledge, this case is the first to include simultaneous complications of pregnancy, a history of tubal ligation, and a paratubal cyst in a surrogate pregnant patient. This case identifies a patient with multiple risk factors whose presentation raised various red flags for emergent diagnosis, including TOA, appendicitis, and adnexal torsion. When imaging results were inconclusive and the patient's symptoms worsened, a diagnostic laparoscopy revealed an adnexal torsion, allowing for surgical resolution with the preservation of the patient's ovary. This case highlights the need for a high clinical suspicion of ovarian torsion in premenopausal and pregnant populations presenting with pelvic pain and an adnexal cyst and to promptly consider surgical intervention when necessary.
